# Organ transplantation and COVID-19

**DOI:** 10.1590/1516-3180.2021.139420052021

**Published:** 2021-06-28

**Authors:** Valter Duro Garcia, Paulo Manuel Pêgo-Fernandes

**Affiliations:** I MD, PhD. Director, Department of Kidney and Pancreas Transplantation, Santa Casa de Misericórdia de Porto Alegre, Porto Alegre (RS), Brazil.; II MD, PhD. Full Professor, Thoracic Surgery Program, Instituto do Coracao, Hospital das Clinicas HCFMUSP, Faculdade de Medicina, Universidade de Sao Paulo, Sao Paulo, SP, BR. Director, Scientific Department, Associação Paulista de Medicina, São Paulo (SP), Brazil.

The pandemic caused by severe acute respiratory syndrome coronavirus 2 (SARS-CoV-2) has transformed the world. Up to May 16, 2021, 162.2 million individuals (2.1% of the world’s population) had become infected, of whom 15.5 million were in Brazil (7.3% of this country’s population), and 3.36 million of these infected individuals had died (432,628 in Brazil).^1^ Thus, in Brazil, the mortality rate up to that date was 2/1,000 of the population and, for 2.8% of the infected individuals, the virus was lethal. This scenario has negatively affected the economy of many countries, causing great losses to thousands of companies, and even bankruptcies; and it has left millions of workers unemployed. The social distancing and isolation that have become necessary to combat the pandemic have harmed social relationships, school activities and non-essential work activities. Meetings and congresses have also been affected. 

Organ transplantation could not remain immune to the coronavirus disease (COVID-19). Organ donation and transplantation activities, and the patients receiving these organs, have also been affected in various ways, as we describe in the following list:

1. Impact on donation and transplantation activities and on waiting lists:
  - Decreased numbers of donors, due to fewer notifications and increased contraindication;
  - Decreased numbers of transplantations, with variation according to the organ transplanted and the type of donor (living or deceased);
  - Decreased entry to waiting lists and increased mortality among patients on these lists;
  - Changes to activities at transplantation centers: with maintenance, diminishment or even temporary suspension of transplantation operations;
  - Modification of outpatient follow-up for transplant patients, such that a large proportion of these patients started to be attended via teleconsultation.
2. Impact on transplant patients:
  - Increased mortality;
  - Increased lethality;
  - Increased morbidity;
  - Diminished immunological response to vaccination.

COVID-19 had an immediate impact on donation and transplantation activities in the countries most affected. In the United States, Spain, France, Netherlands and United Kingdom, there were decreases in donation and transplantation rates of between 36% and 90% during the peak months of the first wave of COVID-19.^2^ This impact on donation and transplantation rates was regional: for example, New York and northern Italy were among the areas most affected, but this impact was temporary. Activities were then resumed as the pandemic subsided in those areas.^3^^,^^4^

In Brazil in 2020, compared with 2019, the donor rate declined by 13%.^5^ This drop in donation rates occurred both through a decrease in notification of potential donors, and through an increase in the number of contraindications for transplantation. The decrease in notification of potential donors could have occurred for any of the following reasons:

- Reduction of the numbers of intensive care unit (ICU) and emergency beds available for potential brain-dead donors because these were occupied by patients with COVID-19;^2^
- Fear, among the population, of taking family members with severe diseases to hospitals, because of the risk of picking up COVID-19, thus leading to increased numbers of deaths at home;
- Reduction of the numbers of cases of head trauma caused either by traffic accidents or by firearms.^2^

The increase in the number of contraindications for transplantation may have occurred through a series of factors, especially at the beginning of the pandemic, including the following:

- Not performing the reverse transcriptase polymerase chain reaction (RT-PCR) test for COVID-19 before removing organs in some places;
- Not having the result from the RT-PCR test for ­COVID-19 before removing organs in other places;
- Potential donors who had been exposed to COVID-19 or who had another respiratory condition, independent of the result from the RT-PCR test for COVID-19, were excluded at the outset of screening.^6^
- Limitations on air transportation leading to diminished interchange of organs between regions because of prolonged cold ischemia times.^7^

The decline in the transplantation rate also varied according to the type of organ: greater for lung and kidney transplantations and smaller for liver and heart transplantations. It also varied according to the type of donor, such that the decline was greater for transplantations with a living donor. Because this is an elective procedure, many such procedures were postponed so as to avoid the risk of donor contamination during the investigation and organ removal.^2^^,^^8^

In Brazil, in 2020, there were declines in the transplantation rates for liver (10%), pancreas (13%), heart (17%), kidney (25%) and lung transplants (39%). The decrease in the number of transplantations with a living donor was greater for kidney transplants (64%) than for liver transplants (13%). There were also variations in the timing of these declines because the pandemic reached different Brazilian states at different times and affected geographical regions differently. Thus, for example, the kidney transplantation rate decreased by 8% in the central-western region and by 80% in the northern region, which has been worst affected by the pandemic.^5^

Analysis on waiting lists is somewhat more complex. On the one hand, with fewer transplantation procedures taking place, there was an accumulation of patients in waiting lists; but on the other hand, the number of patients entering the lists also declined, given that investigations on many patients were postponed.^2^ In addition, in many centers, some of the patients, especially those awaiting transplants of non-vital organs (such as kidney and pancreas transplants), were moved from the active to the inactive category to avoid the most severe phase of the pandemic. In Brazil, for example, declines in the numbers of patients entering the lists, of 18% for liver transplants and 13% for kidney transplants, were observed in 2020. There were also increases in the mortality rates among patients in the lists, of 27% for kidney transplants (going up from 5.2% to 6.6%) and 5% for liver transplants.^5^

Activities in transplantation centers either were maintained or were reduced to transplantations only in special cases, or were temporarily suspended, according to the levels of risk for potential recipients, living donors and professionals; and also according to the conditions in these hospitals.^9^rapidly changing pandemic: consequently, evidence-based recommendations in solid organ transplantation (SOT In Brazil, most kidney transplantation centers in the northern and northeastern regions suspended their activities in the first six months of 2020, while this measure was taken by kidney transplantation centers in the southern region in the third three-month period of the year.

Some societies and organizations relating to transplantation have established recommendations for managing these procedures, including with regard to suspension and resumption of activities and increased safety for recipients and living donors.^2^^,^^9^

Perhaps the only positive legacy from the COVID-19 pandemic in relation to transplantation in Brazil will be the use of telemedicine, with authorization from the Federal Medical Council (Conselho Federal de Medicina, CFM) and reimbursement from the Ministry of Health and health insurance plans for outpatient follow-up of recipients, so as to diminish the risk of transmission of infection to this patient population. This measure has been beneficial not only for preventing transmission of the virus but also for simplifying attending to patients who live in distant locations, and this benefit deserves to be kept after the pandemic.

The first two articles analyzing the impact of COVID-19 on patients who received kidney transplants provided conflicting results. The first case series, from Wuhan, China, published in March 2020, reported on five cases of COVID-19 in kidney transplant patients that occurred in February 2020. These patients presented good evolution, without any deaths. It was concluded that COVID-19 was not severe in this population and that calcineurin inhibitors were capable of blocking the action of SARS-CoV-2.^10^ The second series was published by the transplantation group of Montefiore Hospital, in New York, in April 2020. Thirty-six kidney transplant patients who were affected by COVID-19 in March 2020 were analyzed. Ten of these patients died. It was concluded that the lethality rate among transplant patients (28%) was higher than that of the general population (1%-5%), and higher than that of elderly people over the age of 70 years (8%-15%).^11^

According to some authors, modulation of immunosuppressors may be either harmful or beneficial, depending on the clinical stage of infection in transplant patients. At the start of the infection, strong immunosuppression may adversely affect specific immunity, thus leading to insufficient control over the viral load and prolonged detection of viral ribonucleic acid (RNA) after the onset of the disease. Later on in the course of the disease, immunosuppressive drugs may be beneficial for suppressing pro-inflammatory processes, through maintaining functional inactivation of the immune system.^2^

However, most case series have shown high mortality rates among transplant patients infected by COVID-19. A meta-analysis^12^however, there remains a paucity of robust data in this population. We conducted a systematic review and meta-analysis of SOT recipients with SARS-CoV-2 infection from January 1st t October 9th, 2020. Pooled incidence of symptoms, treatments and outcomes were assessed. Two hundred and fifteen studies were included for systematic review and 60 for meta-analysis. We identified 2,772 unique SOT recipients including 1,500 kidney, 505 liver, 141 heart and 97 lung. Most common presenting symptoms were fever and cough in 70.2% and 63.8% respectively. Majority (81% on 37 articles about COVID-19 in transplant patients showed that the mortality rate was 18.6%. In an article from the Kidney Hospital (Hospital do Rim) in São Paulo, 11,875 kidney transplant recipients who were followed up as outpatients were analyzed. Among these patients, 491 became infected with COVID-19 and the mean lethality rate was 28.5%, ranging from 6% among young patients without comorbidities to 41% among elderly patients with comorbidities. This demonstrated that immunosuppression, age and comorbidities are important risk factors in this population. In the same study, it was observed that among the patients who recovered from COVID-19, 19% presented permanent dysfunction of the graft and 4%, loss of the graft.^13^

Transplantation in infected candidates and use of organs from donors with COVID-19 are currently only recommended after resolution of the clinical symptoms and with a negative RT-PCR result. Nonetheless, cases of inadvertent transplantation of organs from asymptomatic donors who showed positive RT-PCR results, without transmission to the recipient, have been reported.^14^ This may indicate future potential for using donors who are RT-PCR-positive, for life-saving procedures, especially after gaining greater knowledge about the correlation between RT-PCR positivity and infectiveness.^2^ There is only one report on transmission from a lung donor infected with COVID-19 to the recipient of that organ.^14^

Initial studies on vaccination against COVID-19 among kidney transplant patients have shown that the immunological response in lower among these individuals, as already seen with vaccination against influenza viruses. In two studies, with 436 and 242 transplant patients, only 17% and 11% presented a serological response, measured 20 and 28 days after the first dose of messenger RNA (mRNA) vaccine.^15^^,^^16^1,2 a low proportion (17% Continuation of these studies^17^^,^^18^ showed that after the second dose of these vaccines, in 658 and 205 patients, a serological response was seen in 54% and 48%, while in the control group it was 100%. In three studies conducted in Israel,^19^^,^^20^^,^^21^ among kidney (136), liver (80) and lung transplant patients (180), a serological response was observed in 37.5%, 47.5% and 18%, respectively.

These data, if confirmed by other studies, suggest that alternative strategies need to be used in relation to these patients, such as greater numbers of doses; or use of mycophenolate suspension for some days before and after vaccination; or a temporary change to azathioprine or mammalian target of rapamycin (mTOR) inhibitor. These seem to present a better serological response to the vaccine, but this needs to be confirmed.

Thus, organ transplantation has also been dramatically affected by COVID-19, with regard both to transplantation activities and to the severity of cases (with elevated morbidity and mortality) and poor response to vaccination among transplant patients. 

## References

[1] World Health Organization (2021). WHO Coronavirus (COVID-19) Dashboard. WHO Coronavirus (COVID-19) Dashboard With Vaccination Data [Internet].

[2] Danziger-Isakov L, Blumberg EA, Manuel O, Sester M (2021). Impact of COVID-19 in solid organ transplant recipients. Am J Transplant.

[3] Angelico R, Trapani S, Manzia TM (2020). The COVID-19 outbreak in Italy: Initial implications for organ transplantation programs. Am J Transplant.

[4] Cholankeril G, Podboy A, Alshuwaykh OS (2020). Early impact of COVID-19 on solid organ transplantation in the United States. Transplantation.

[5] Registro Brasileiro de Transplantes (RBT) Dimensionamento dos Transplantes no Brasil e em cada estado (2013-2020). https://site.abto.org.br/wp-content/uploads/2021/03/rbt_2020_populacao-1-1.pdf.

[6] Domínguez-Gil B, Coll E, Fernández-Ruiz M (2020). COVID-19 in Spain: Transplantation in the midst of the pandemic. Am J Transpl.

[7] Strauss AT, Cartier D, Gunning BA (2020). Impact of the COVID-19 pandemic on commercial airlines in the United States and implications for the kidney transplant community. Am J Transplant.

[8] Lentine KL, Vest LS, Schnitzler MA (2020). Survey of US Living Kidney Donation and Transplantation Practices in the COVID-19 Era. Kidney Int Reports.

[9] Boyarsky BJ, Po-Yu Chiang T, Werbel WA (2020). Early impact of COVID-19 on transplant center practices and policies in the United States. Am J Transplant.

[10] Zhang H, Chen Y, Yuan Q (2020). Identification of Kidney Transplant Recipients with Coronavirus Disease 2019. Eur Urol.

[11] Akalin E, Azzi Y, Bartash R (2020). Covid-19 and Kidney Transplantation. N Engl J Med.

[12] Raja MA, Mendoza MA, Villavicencio A (2021). COVID-19 in solid organ transplant recipients: A systematic review and meta-analysis of current literature [Internet]. Transplant Rev (Orlando).

[13] Cristelli MP, Viana LA, Dantas MTC (2021). The Full Spectrum of COVID-19 Development and Recovery Among Kidney Transplant Recipients. Transplantation.

[14] Hong HL, Kim SH, Choi DL, Kwon HH (2020). A case of coronavirus disease 2019–infected liver transplant donor. Am J Transplant.

[15] Boyarsky BJ, Werbel WA, Avery RK (2021). Antibody Response to 2-Dose SARS-CoV-2 mRNA Vaccine Series in Solid Organ Transplant Recipients. JAMA.

[16] Benotmane I, Gautier-Vargas G, Cognard N (2021). Weak anti–SARS-CoV-2 antibody response after the first injection of an mRNA COVID-19 vaccine in kidney transplant recipients. Kidney Int.

[17] Boyarsky BJ, Werbel WA, Avery RK (2021). Immunogenicity of a Single Dose of SARS-CoV-2 Messenger RNA Vaccine in Solid Organ Transplant Recipients [Internet]. JAMA.

[18] Benotmane I, Gautier-Vargas G, Cognard N (2021). Low immunization rates among kidney transplant recipients who received 2 doses of the mRNA-1273 SARS-CoV-2 vaccine. Kidney Int.

[19] Grupper A, Rabinowich L, Schwartz D (2021). Reduced humoral response to mRNA SARS-Cov-2 BNT162b2 vaccine in kidney transplant recipients without prior exposure to the virus. Am J Transplant.

[20] Rabinowich L, Grupper A, Baruch R (2021). Low immunogenicity to SARS-CoV-2 vaccination among liver transplant recipients. J Hepatol.

[21] Shostak Y, Shafran N, Heching M (2021). Correspondence Early humoral response among lung transplant recipients vaccinated with BNT162b2 vaccine. Lancet Respir Med.

